# Walking With Leg Blood Flow Restriction: Wide-Rigid Cuffs vs. Narrow-Elastic Bands

**DOI:** 10.3389/fphys.2020.00568

**Published:** 2020-05-29

**Authors:** Sten Stray-Gundersen, Savannah Wooten, Hirofumi Tanaka

**Affiliations:** Cardiovascular Aging Research Laboratory, Department of Kinesiology and Health Education, The University of Texas at Austin, Austin, TX, United States

**Keywords:** exercise, cardiovascular disease, exercise physiology, exercise training, physical exercise, hemodynamic stress

## Abstract

**Background:**

Blood flow restriction (BFR) training is becoming a popular form of exercise. Walking exercise in combination with pressurized wide-rigid (WR) cuffs elicits higher cardiac workload and a vascular dysfunction due presumably to reperfusion injury to the endothelium. In contrast, narrow-elastic (NE) BFR bands may elicit different hemodynamic effects. Therefore, we compared the acute cardiovascular responses to two distinct forms of BFR training during light-intensity exercise.

**Methods and Results:**

15 young healthy participants (M = 9, F = 6) performed five bouts of 2-min walking intervals at 0.9 m/s with a 1-min rest and deflation period with either WR, NE, or no bands placed on upper thighs. Cuff pressure was inflated to 160 mmHg in WR cuffs and 300 mmHg in NE bands while no cuffs were used for the control. Increases in heart rate and arterial blood pressure were greater (*p* < 0.05) in the WR than the NE and control conditions. Double product increased to a greater extent in the WR than in the NE and control conditions. Increases in perceived exertion and blood lactate concentration were greater (*p* < 0.05) in the WR compared with the NE and control conditions (*p* < 0.05), while no differences emerged between the NE and control conditions. There were no changes in arterial stiffness or brachial artery flow-mediated dilation (FMD) after all three trials.

**Conclusion:**

Use of WR BFR cuffs resulted in a marked increase in blood pressure and myocardial oxygen demand compared with NE BFR bands, suggesting that NE bands present a safer alternative for at-risk populations to perform BFR exercise.

**Clinical Trial Registration:**

This study was registered in the Clinicaltrials.gov (NCT03540147).

## Introduction

Over the past two decades, blood flow restriction (BFR) training has increased in popularity among athletes, researchers, and physical therapists (Pope et al., 2013). During this form of training, users place pressurized cuffs/bands or non-pressurized straps/wraps on the most proximal portion of the limb in order to restrict venous blood flow while maintaining varying degrees of arterial inflow (Loenneke et al., 2013). The restriction of venous blood flow while performing light-weight exercises leads to venous pooling and local metabolic changes that together stimulate systemic adaptations similar to those achieved with heavy exercise (Takarada et al., 2000; Fujita et al., 2008). Since BFR used in combination with low-intensity walking exercise can confer significant improvements in muscle strength and hypertrophy (Takarada et al., 2000; Abe et al., 2005), there is great potential for use with clinical populations for fitness and rehabilitation.

Concern has been raised over the use of BFR in at-risk populations (e.g., hypertensive, obese, atherosclerotic) due to the potential for deep vein thrombosis, rhabdomyolysis, pulmonary emboli (Nakajima et al., 2006; Yasuda et al., 2017), and other serious complications associated with occluding arterial flow and performing skeletal muscle contractions. One such complication could be an augmentation of the exercise pressor reflex, which is exaggerated in certain at-risk populations (Manisty and Francis, 2007), and is normally elicited during exercise by the stimulation of group III and IV afferents (local mechano- and metaboreceptors), resulting in a sympathetically mediated elevation in blood pressure and heart rate. Since BFR training leads to an accumulation of metabolites and exerts high pressures on blood vessels and contracting muscle tissue, it seems likely to elicit an exaggerated blood pressure response (Spranger et al., 2015). Indeed, wide-rigid (WR) BFR cuffs can cause painful compression of tissues, increases in systemic vascular resistance, acute vascular dysfunction, and increased myocardial demand even at low exercise intensities (Renzi et al., 2010; Sugawara et al., 2015).

However, a multitude of Japanese athletes, seniors, clinicians, and trainers have been using BFR in the form of Kaatsu for over 30 years with an extremely low incidence of serious complications (Nakajima et al., 2006; Yasuda et al., 2017). The more recent findings and resulting concerns may be due to a shift from the original narrow-elastic (NE) design present in the Kaatsu bands to WR nylon cuffs adapted from surgical tourniquets and blood pressure cuffs. The WR cuffs are easily available, but have the potential to inhibit the expansion of muscle upon increased blood flow accompanying exercise and muscle contraction while the NE bands do not prevent the expansion.

To elucidate the potentially differing effects of these two distinct forms of BFR, we assessed the acute hemodynamic responses of 15 young healthy individuals during low-intensity walking exercise while using WR cuffs or NE bands. We hypothesized that the WR cuffs would elicit greater pressor responses and myocardial oxygen demand compared with the NE bands during light-intensity aerobic exercise. Additionally, we hypothesized that systemic endothelial function and arterial stiffness will not be affected by an acute bout of BFR walking exercise, regardless of cuff type. If discovered that the NE bands do not elicit the same heightened blood pressure responses to BFR, they may present a safer alternative for at-risk populations predisposed to exhibit exaggerated hemodynamic responses during exercise. We studied young healthy adults as they are the biggest users of BFR and to first determine whether the hemodynamic responses observed were within safe ranges before extending the studies to more vulnerable populations.

Blood flow restriction using WR cuffs may induce an ischemia-reperfusion injury to the distal vessels upon the release of ischemia (Renzi et al., 2010). One of the hallmark features of the ischemia-reperfusion injury is endothelial dysfunction leading to arterial stiffening. These vascular changes induced by the BFR exercise could be unfavorable or even detrimental to those with compromised cardiovascular conditions. Indeed our previous investigation (Renzi et al., 2010) found a reduction in popliteal FMD after performing submaximal walking bouts with WR cuffs placed on thighs. Accordingly, we determined whether this effect was systemic in nature or localized to the occluded artery by assessing brachial endothelial function after the BFR exercise with cuffs placed on thighs.

## Materials and Methods

### Participants

A total of 15 young healthy sedentary and recreationally active adults (nine males and six females) between the ages of 18 and 35 years participated in this study. Exclusion criteria for participation, assessed via medical history questionnaire, included (1) uncontrolled hypertension; (2) smoking within the last 6 months; (3) a history of heart disease, kidney disease, peripheral artery disease, and other known cardiovascular issues; (4) obesity as defined by a body mass index (BMI) > 30 kg/m^2^; (5) a history of diabetes or other metabolic dysfunction; (6) major operations within the last 6 months; (7) advised to avoid exercise by a physician; (8) part of a vulnerable population (unable to consent, pregnant women, osteoporotic, etc.): or (9) currently performing BFR training. All participants submitted their written informed consent prior to participation. The Institutional Review Board reviewed and approved this study.

### Procedures

Participants visited the laboratory on two separate occasions for 2 h per visit. During the first visit, anthropometric measures of height, body weight, and body fatness were taken. Body fatness was estimated using the seven-site skinfold technique with Lange skinfold calipers (Beta Technology, Santa Cruz, CA, United States). Participants fasted for at least 8 h, did not consume alcohol or caffeine for 12 h, and abstained from strenuous exercise for 24 h prior to each experimental session.

After 20 min of supine rest in a quiet, temperature-controlled room (23–27°C), baseline measurements, including heart rate, blood pressure using an automated sphygmomanometer, arterial stiffness using a pulse-wave velocity index, and brachial endothelial function via flow-mediated dilation (FMD), were conducted. After undergoing baseline measurements, each participant performed one of three randomly assigned walking exercise conditions; walking with pressurized WR cuffs, walking with pressurized NE bands, or walking without cuffs/bands (control). Cuffs/bands were placed on both legs and subsequently inflated when performing one of the BFR conditions while no cuffs were used when performing the control. In men, visits were separated by at least 3 days. In women, visits were scheduled ∼1 month apart during the early follicular phase of the menstrual cycle approximately 1–5 days following the start of menstruation. All participants performed the three conditions in a randomized order on three separate days. In addition to taking plasma lactate samples immediately before and after exercise, we assessed RPE before, mid-way through, and after exercise. During the exercise, we recorded beat-by-beat blood pressure and heart rate continuously via finger plethysmography. Once the participant completed the exercise, measures of vascular functions were repeated within a 15-min period and then 1 h after the completion of the exercise.

### Measurements

Heart rate at rest, brachial blood pressure, and arterial stiffness as assessed by cardio-ankle vascular index (CAVI) were measured in the supine position using the automated vascular screen device (VaSera, Fakuda Denshi, Tokyo, Japan) as previously described (Lim et al., 2016).

Flow-mediated dilation, a measure of vascular endothelium-dependent vasodilation, was assessed using a semi-automated diagnostic ultrasound system with a semi-automated probe, which self-adjusts to provide clear images of the intimal layer for baseline artery diameter measurements (EF-38G, UNEX corporation, Nagoya, Japan) (Fico et al., 2019). While participants rested in the supine position, a pneumatic cuff was placed on the right forearm. Then cross-sectional and longitudinal images of the brachial artery were acquired 6–8 cm proximal to the cuff. In order to occlude blood flow, the cuff was subsequently inflated to 50 mmHg above resting systolic blood pressure for a period of 5 min. Upon cuff deflation, blood flow velocity and artery diameter were measured for an additional 2 min.

Blood lactate concentration was measured immediately before and between 90 and 120 s after the walking protocol. Using disposable lancets, we punctured the finger-tip and collected a blood droplet on a disposable lactate test-strip. We did not warm the fingers prior to the finger-prick as it was not necessary for this population. All blood samples were analyzed using a portable lactometer (LactatePro, Arkray; Kyoto, Japan).

Ratings of perceived exertion were assessed before, during, and after the walking protocol. Participants were familiarized with the scale prior to the beginning of the test and asked to score their perceived exertion using the original Borg scale.

In order to record hemodynamics during the walking protocol, beat-to-beat arterial blood pressure waveforms were continuously measured via finger plethysmography (Portapres Model 2, TNO TPD Biomedical Instruments, Netherlands) placed on the middle finger of the left hand of each participant. Following standard procedure in order to control for potential changes in hydrostatic pressure due to variable hand position, participants were instructed to keep the left hand at heart level during the entirety of the exercise session. The participant’s right hand was free to move in a normal walking fashion or to stabilize themselves during a trip or fall. Heart rate was calculated from the finger blood pressure waveform using the validated model-flow method (BeatScope 1.0 software, TNO TPD Biomedical Instrumentation, Amsterdam, Netherlands). Double product, an index of myocardial oxygen demand, was calculated by systolic blood pressure × heart rate. The hemodynamic values represent the average values during the 2-min walking bout, excluding the 1-min rest interval.

### Exercise Protocol

The walking exercise test consisted of five bouts of 2-min walking intervals at 0.9 m/s with a 1-min rest and deflation period between each bout with either WR cuffs, NE bands, or no cuffs placed on the upper thighs (Abe et al., 2006; Renzi et al., 2010; Sugawara et al., 2015). The chosen treadmill speed has been used in previous investigations in our laboratory as a means to evoke a submaximal effort, and is a typical speed used during cardiac rehabilitation programs (Abe et al., 2006; Renzi et al., 2010; Sugawara et al., 2015). We used two commercially available cuffs as representatives of WR cuffs and NE bands. For the WR cuff condition, we used wide rapid-inflation pneumatic tourniquets (Hokanson, CC17, Bellevue, WA, United States; 18 cm wide × 108 cm long). We used a one-size-fits-all thigh-cuff typically used by people performing lower limb BFR, and did not observe any differences in responses between smaller and larger participants. For the NE band condition, we used pneumatically-controlled BFR leg bands (BStrong, Park City, UT, United States; 5 cm wide × 50 cm long). Following previous protocols (Abe et al., 2006; Renzi et al., 2010), and in order to familiarize the participant with the WR cuff, we initially inflated the cuff to 120 mmHg for 30 s, released it for 10 s, re-inflated to 140 mmHg for 30 s, released for 10 s, and then re-inflated to the final pressure of 160 mmHg. Standing baseline heart rate and blood pressure was recorded via finger plethysmography for 1-min before beginning the walking exercise. For the NE condition, we gradually inflated the bands to 300 mmHg, which is the recommended and commonly used pressure for leg BFR according to the company supplying the equipment. These same standard pressures were used for all individuals for comparative purposes. Once the desired pressure was reached, the participants began the walking exercise. After completion of each 2-min bout, we rapidly deflated both cuffs for 1-min before the next bout. After the fifth bout, we continued recording blood pressure and heart rate for one additional minute. In the control session, participants performed the same exercise protocol without the application or inflation of either cuff.

### Statistical Analyses

Parametric statistics were used as the data were normally distributed as determined by a Levene’s test. Since baseline measures for systemic hemodynamics were not different, one-way repeated measures ANOVA was used to identify significant effects across the three conditions. For ratings of perceived exertion, blood lactate, CAVI, and FMD, two-way repeated measures ANOVA was used. After determining whether significant main effects or interactions (*p* < 0.05) were present, we ran *post hoc* multiple comparison *t*-tests (*p* < 0.05) using a Bonferroni correction to identify the significant differences between conditions. Data are presented as means ± SEM unless stated otherwise.

## Results

**FIGURE 1:**
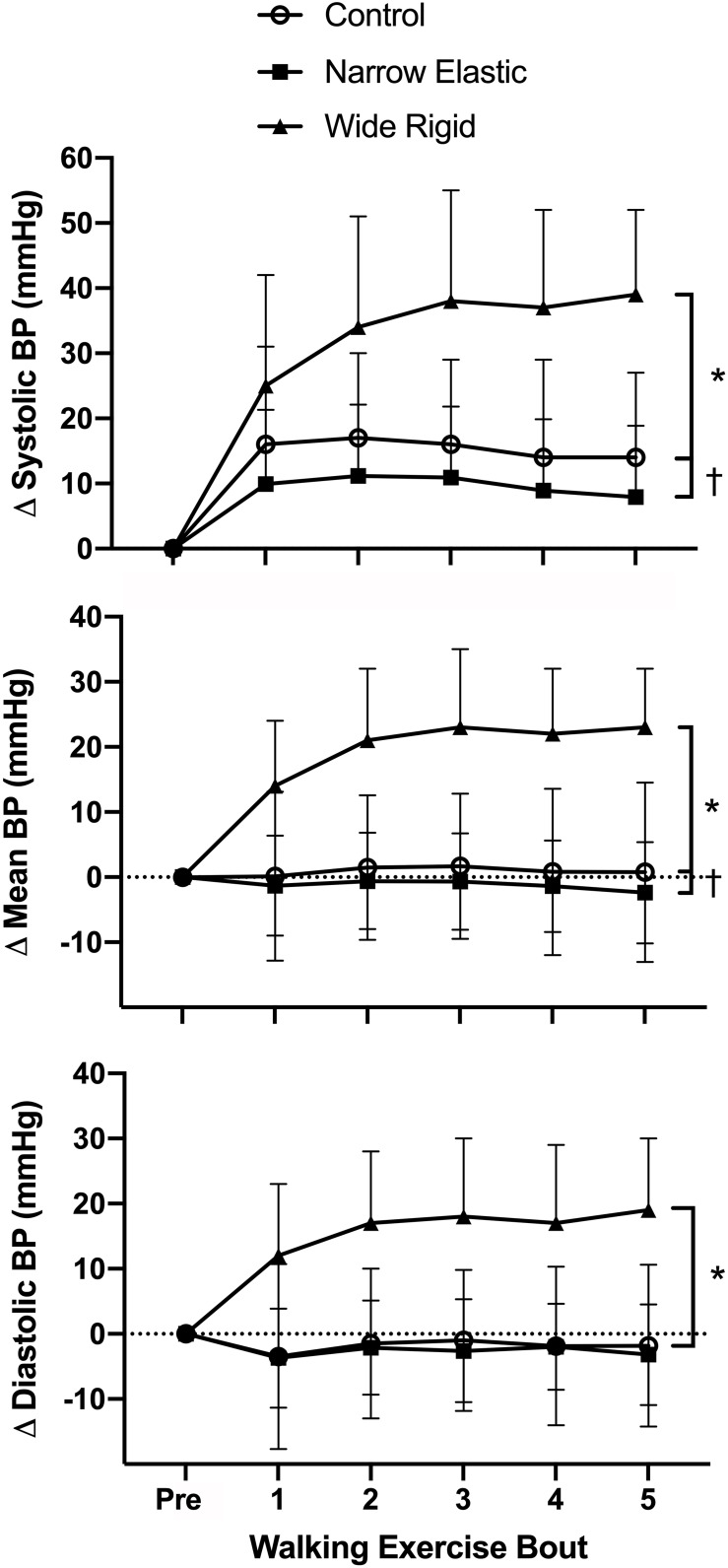
Systolic, mean, and diastolic arterial blood pressure (BP) before and during each 2-min interval of the walking exercise. *P < 0.05 vs. Wide Rigid cuffs, ^†^*P* < 0.05 vs. the Control. Data are means ± SEM.

**FIGURE 2:**
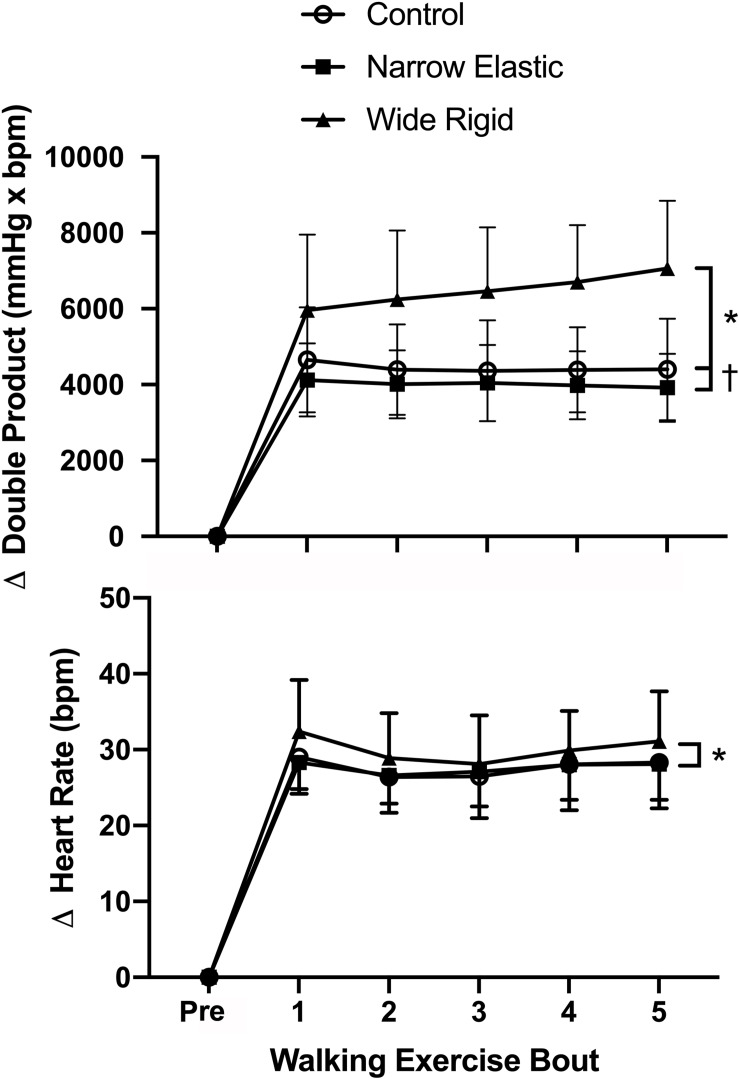
Changes in double product and heart rate before and during each 2-min interval of the walking exercise. *P < 0.05 vs. Wide Rigid cuffs, ^†^*P* < 0.05 vs. Control. Data are means ± SEM.

**FIGURE 3:**
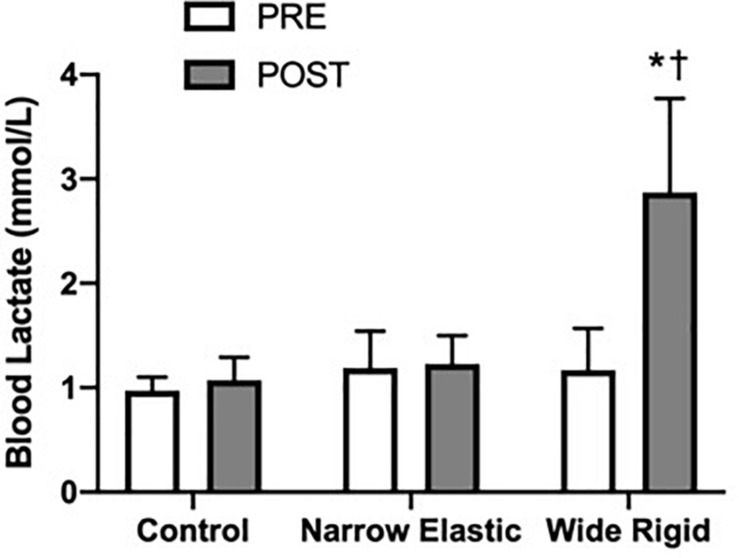
Blood lactate concentrations before and immediately after the walking exercise. *P < 0.05 vs. Pre, ^†^*P* < 0.05 vs. the control. Data are means ± SEM.

**FIGURE 4:**
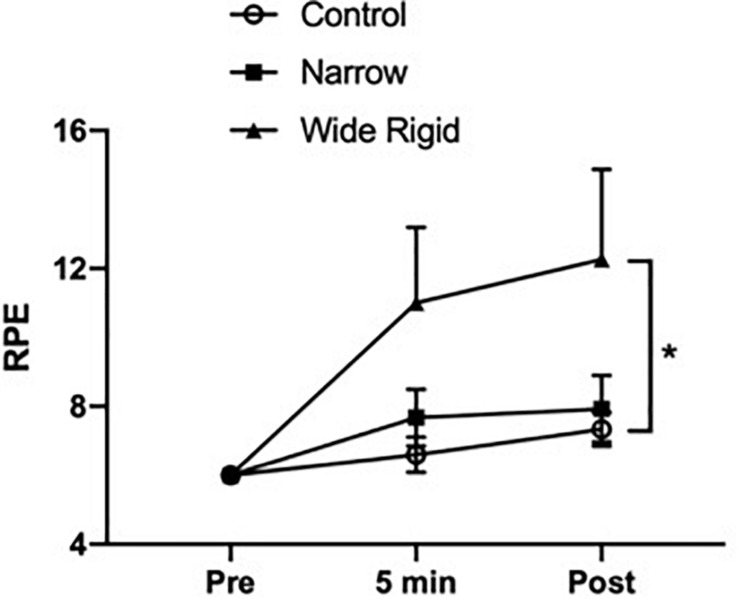
Ratings of perceived exertion (RPE) before (Pre), 5 min into the exercise, and immediately after (Post) the walking exercise. *P < 0.05 vs. Pre. Data are presented as means ± SEM.

**FIGURE 5:**
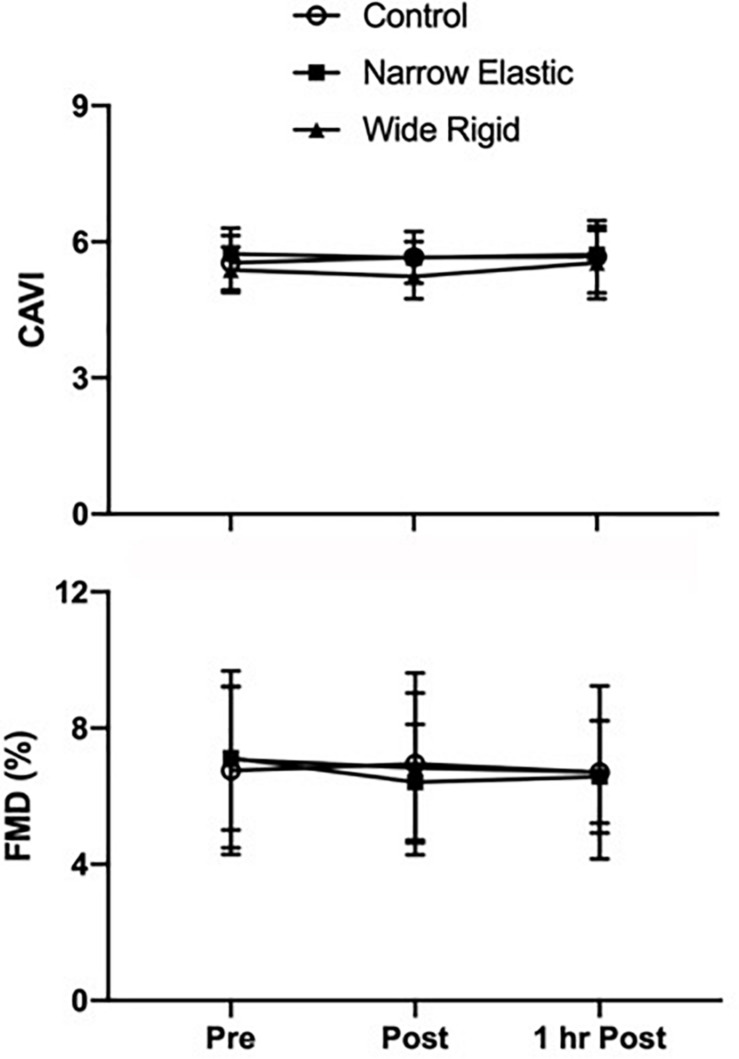
Cardio-ankle vascular index (CAVI) and flow-mediated dilation (FMD) assessed before (Pre), immediately after (Post), and 1 h after (1HR Post) walking exercise. Data are presented as means ± SEM.

Selected participant characteristics are presented in Table 1. Participants were young, healthy, and exhibited normal body weight and composition. Absolute values for selected hemodynamic variables before and during each 2-min walking bout are presented in Table 2. Changes in arterial blood pressure from baseline during each 2-min walking bout are presented in Figure 1. At baseline, no differences existed in any of the variables between the three conditions. Increases in blood pressure were greater (*p* < 0.05) in the WR cuff condition than the NE band and control conditions while increases in systolic and mean arterial blood pressure were greater in the control compared with the NE condition (*p* < 0.05). As presented in Figure 2, increases in double product were greater (*p* < 0.05) in the WR cuff condition than the NE band and control conditions and increases were greater in the control compared with the NE condition (*p* < 0.05). Increases in heart rate were greater (*p* < 0.05) in the WR condition than the NE and control conditions and were not different between the NE condition and control (*p* > 0.05). Blood lactate concentrations measured before and immediately after walking are presented in Figure 3. Increases in blood lactate concentrations were greater (*p* < 0.05) in the WR cuff condition than the NE band and control conditions. As shown in Figure 4, ratings of perceived exertion were greater (*p* < 0.05) in the WR condition than the control condition immediately post-exercise. CAVI and FMD did not change across all three conditions (Figure 5).

**TABLE 1:** Selected participants’ characteristics.

Variable	Mean ± SEM or *n*
Age (years)	23 ± 2
Sex	9M/6F
Height (cm)	174 ± 10
Body weight (kg)	70 ± 14
Body mass index (kg/m^2^)	23 ± 3
Body fat (%)	17 ± 5

*No sex-based or race/ethnicity-based differences were present.*

**TABLE 2:** Cardiovascular variables before (Pre) and during (During) an acute bout of walking exercise.

	Control (*n* = 15)		Narrow elastic (*n* = 15)		Wide rigid (*n* = 15)	
Variable	Baseline	Walking	Baseline	Walking	Baseline	Walking
Systolic BP (mmHg)	115 ± 8	130 ± 15	116 ± 9	127 ± 10	116 ± 11	150 ± 16^*†^
Diastolic BP(mmHg)	69 ± 8	66 ± 13	67 ± 7	66 ± 12	66 ± 7	85 ± 15^*†^
Mean BP (mmHg)	84 ± 7	85 ± 13	84 ± 7	86 ± 9	82 ± 7	108 ± 17^*†^
Double product (mmHg × bpm)	6427 ± 1130	10,904 ± 1543^*^	6488 ± 934	10,526 ± 1011^*^	6984 ± 1472	13,296 ± 2017^*†^
Heart rate (bpm)	56 ± 9	84 ± 8^*^	56 ± 6	83 ± 7^*^	60 ± 9	88 ± 7^*†^

BP = blood pressure. * *P* < 0.05 from Pre, ^†^*P* < 0.05 from control; data are means ± SEM.

## Discussion

The present study aimed to evaluate hemodynamic responses between two distinct forms of BFR training commonly used by trainers, physical therapists, and researchers. In agreement with previous investigations (Renzi et al., 2010; Sugawara et al., 2015), the use of WR BFR cuffs elicited markedly increased blood pressure responses and heightened myocardial oxygen demands during light intensity walking compared with the NE bands and control conditions. In contrast, the use of NE bands did not elicit increased hemodynamic responses compared to control, suggesting that NE BFR does not appear to pose additional risk to users than light-intensity walking without BFR. In fact, systolic blood pressure, mean arterial blood pressure, and double product values were greater in the control condition compared with the NE condition. None of the conditions induced acute measurable changes in cardio-ankle vascular indices or FMD, suggesting that these forms of BFR do not promote systemic vascular dysfunction or arterial stiffening. These findings are novel and suggest that NE BFR may present a safe option for at-risk populations to perform BFR as a mode of exercise and rehabilitation.

The mechanisms underlying the differing hemodynamic and metabolic responses to the two forms of BFR exercise remain elusive and beyond the scope of this investigation as these variables were not measured in the present study due to technical issues. However, it is clear that the width and material of the cuff have profound effects on systemic hemodynamics. This is likely due to varying degrees of arterial occlusion and compression of muscle tissue leading to variable increases in blood pressure, systemic vascular resistance, and local mechanoreceptor stimulation (Loenneke et al., 2013; Spranger et al., 2015). In particular, the use of WR cuffs results in highly compressive forces over a large area of the limb, inhibiting the muscle from expanding with an increase in blood flow accompanying exercise. In contrast, an NE design appears to minimize how much working muscle is compressed during repeated muscle contractions, allowing the muscle to swell upon increased blood flow. This is evidenced by the slight drop in diastolic blood pressure observed during the control and NE conditions. It appears that NE BFR systems provide a wide range of pressures in which one can avoid arterial occlusion, while sufficiently restricting venous outflow to create a disturbance of homeostasis in working muscle. With WR BFR systems, due to the rigid outer material that cannot expand, the cuff is not able to accommodate the increase in cross-sectional area when muscle contracts and increases its cross-sectional area—pressures spike in the tissues contained by the cuff, arteries become occluded, and veins remain closed. Thus, the WR BFR system stops functioning correctly and complications like peripheral nerve injury, rhabdomyolysis, and deep venous thrombi (DVTs) become more likely.

The elastic nature of bands enables the distal portion of the muscle to force blood past the intermittent venous blockade, minimizing the degree of pain, arterial occlusion, and mechanical compression of tissues. Furthermore, since BFR can be equally effective at inducing hypertrophy and strength gains at 40 and 90% arterial occlusion with the use of WR cuffs (Counts et al., 2016), the percent arterial occlusion does not appear to be of primary concern for an effective BFR session. This is of considerable importance given that an increase in systemic vascular resistance, which is elevated when occluding any percentage of arterial inflow, leads to increases in blood pressure and heart rate (Spranger et al., 2015). In contrast, during aerobic exercise of varying intensities, systemic vascular resistance decreases slightly due to a vasodilatory response in the working muscle to exercise (Laughlin, 1999). Therefore, the observed increases in systemic vascular resistance during BFR (Renzi et al., 2010; Pinto and Polito, 2016) are likely a result of the mechanical constriction of muscle and other tissues beneath the cuff that functionally inhibits tissue expansion and reduces the effect of the local vasodilatory response elicited during aerobic exercise. When using NE bands, this mechanically mediated rise in systemic vascular resistance appears to be absent as muscle contractions are able to intermittently pump blood past the venous impediment and induce peripheral vasodilation. This is primarily evidenced by the considerable increase in diastolic pressure while using the WR cuffs compared with the NE and control conditions. This suggests that the exercise pressor reflex only becomes exaggerated when using WR cuffs at a commonly used pressure of 160 mmHg, prompting the need for individualized pressures and/or use of NE bands with at-risk populations.

The lower systolic and mean blood pressure in the narrow elastic band condition compared with the control condition that we observed in the present study is perplexing. In the present study, heart rate was not different between the NE band and the control conditions. Since systolic blood pressure is driven by changes in stroke volume (Tanaka et al., 2016), it is possible that the NE band somehow facilitated venous blood pooling, which in turn reduced venous return, preload, stroke volume resulting in lower systolic blood pressure. Indeed the previous investigation (Renzi et al., 2010) found that increases in stroke volume were attenuated in the BFR condition compared with the control.

In addition to exaggerated hemodynamic responses, the WR cuffs elicited significantly higher ratings of perceived exertion and increases in plasma lactate concentrations. This suggests a greater accumulation of metabolites in the muscle during the WR condition than the NE and control conditions given the same absolute workload. Although speculative, this could be due to a greater degree of arterial impediment leading to more anaerobic metabolism, and more venous blood pooling as the skeletal muscle pump is unable to push metabolite-rich blood past the occluding cuff. This may be useful for healthy individuals performing low loads in a highly controlled environment such as a physical therapist clinic. However, it also poses the risk of an augmentation of the exercise pressor reflex, leading to unnecessary increases in blood pressure to achieve the desired BFR stimulus.

In agreement with a past investigation (Rossow et al., 2012), participants frequently complained of pain from the compression of the cuff when using the WR cuffs while there were no complaints when using the NE bands. This may have confounded the ratings of perceived exertion seen in the WR condition as pain can augment relative measures of effort during certain types of exercises (Hollander et al., 2003). However, pain also stimulates sympathetic activity, which could lead to an even greater augmentation of the exercise pressor reflex (Spranger et al., 2015). Therefore, more investigation into the use of NE bands during intense exercise, and the subsequent increase in muscle pain is warranted to determine the mechanism responsible for the differences observed.

In a previous study (Renzi et al., 2010), walking in combination with WR cuffs acutely decreased endothelial function as assessed via FMD of the popliteal artery. To determine whether the acute endothelial dysfunction previously observed was localized to the vasculature that was exposed to ischemia and reperfusion or a sign of systemic endothelial dysfunction, we measured brachial artery diameter changes in response to post-occlusive reactive hyperemia. The FMD values remained unchanged across all conditions and time points, suggesting that the acute decrease in popliteal FMD previously observed was likely a consequence of local ischemia-reperfusion injury to the vascular endothelium distal to the cuff, and not a drop in systemic endothelial function.

There were several limitations to this study. As the purpose of this study was to acutely determine the relative safety among different forms of BFR in young healthy individuals, there was no measure of the degree of efficacy between cuffs. Additionally, the responses of young healthy individuals do not necessarily translate to more vulnerable populations. Moreover, since this study only assessed acute responses to walking exercise with BFR, we cannot definitely say whether these responses would be the same for long-term aerobic or resistance training. Clearly, there is a need for further research investigating the cardiovascular effects of various forms of BFR and exercise protocols.

There are several opportunities to further elucidate the mechanisms for the observed responses in the present study as well as the use of BFR in various populations and protocols. First, as the present study assessed differences between WR and NE cuffs, further research showing the potential differences between narrow-rigid and wide-elastic cuffs are needed to further elucidate the cause of the increased pressor response. Although hemodynamic responses to various forms of BFR have been investigated at rest (Loenneke et al., 2015), the same measures need to be conducted during exercise. In addition, there is a need for more research on BFR as a rehabilitation tool for hypertensive patients. Although several researchers have found evidence of an attenuation of hypertension after a BFR training program (Cezar et al., 2016; Pinto and Polito, 2016; Shimizu et al., 2016; Vanwye et al., 2017), more investigation into the precise mechanism as well as the use of a variety of training protocols is necessary. There is a spectrum of BFR equipment that can elicit the desired stimulus at various pressures and intensities (Wilk et al., 2018), so individually determining the optimal type of equipment and exercise protocols could provide opportunities for a wide range of the population to use BFR.

## Conclusion

The main finding in the present study is that the use of WR BFR cuffs elicited markedly increased pressor responses and a heightened myocardial oxygen demand during low-intensity walking compared with the NE bands or control. It appears that an exaggerated blood pressure response should be expected when using WR BFR cuffs that increase systemic vascular resistance by occluding arterial inflow, compressing tissues, and reducing the ability of the skeletal muscle pump to function. Therefore, we conclude that WR cuffs may only be safe within a narrow window of pressures and should be conducted in a setting in which continuous hemodynamics are monitored. In contrast, the NE bands do not seem to elicit an augmented exercise pressor response compared to control. These findings suggest that at-risk populations can perform BFR without fear of overt cardiovascular risk. By nature of its width and material, it is difficult to minimize the risks associated with WR cuffs when occluding any amount of arterial blood flow, and as such, it should be prescribed carefully.

## Data Availability Statement

The raw data supporting the conclusions of this article will be made available by the authors, without undue reservation, to any qualified researcher.

## Ethics Statement

The studies involving human participants were reviewed and approved by IRB at the University of Texas at Austin. The patients/participants provided their written informed consent to participate in this study.

## Author Contributions

SS-G was responsible for data collection, data analysis, authoring of the manuscript, and generation of figures and tables. SW aided in data collection, data acquisition, and data analysis. HT was responsible for study design, overseeing the entirety of the project, editing and reviewing the manuscript, and provided final formatting of the document.

## Conflict of Interest

A conflict of interest was declared by SS-G, who is presently employed by BStrong, Park City, UT, United States. The remaining authors declare that the research was conducted in the absence of any commercial or financial relationships that could be construed as a potential conflict of interest.
